# Sinonasal Outcomes Obtained after 2 Years of Treatment with Benralizumab in Patients with Severe Eosinophilic Asthma and CRSwNP: A “Real-Life” Observational Study

**DOI:** 10.3390/jpm14091014

**Published:** 2024-09-23

**Authors:** Eugenio De Corso, Dario Antonio Mele, Angela Rizzi, Camilla Spanu, Marco Corbò, Serena Pisciottano, Rodolfo Francesco Mastrapasqua, Silvia Baroni, Davide Paolo Porru, Gabriele De Maio, Alberta Rizzuti, Giuseppe Alberto Di Bella, Augusta Ortolan, Matteo Bonini, Francesca Cefaloni, Cristina Boccabella, Francesco Lombardi, Raffaella Chini, Cristiano Caruso, Marco Panfili, Jacopo Galli

**Affiliations:** 1 UOC Otorinolaringoiatria, Fondazione Policlinico Universitario Agostino Gemelli—IRCCS, 00168 Roma, Italy; eugenio.decorso@policlinicogemelli.it (E.D.C.); jacopo.galli@policlinicogemelli.it (J.G.); 2 Department of Head-Neck and Sensory Organs, Università Cattolica del Sacro Cuore, 00168 Rome, Italy; camillaspanu@gmail.com (C.S.); marco.corbo@icloud.com (M.C.); serena.pisciottano01@icatt.it (S.P.); davide.cp@tiscali.it (D.P.P.); gabrieledemaio1@gmail.com (G.D.M.); albrizzuti@libero.it (A.R.); giuseppealberto.dibella@gmail.com (G.A.D.B.); 3 Unit of Allergology e Clinical Immunology, Fondazione Policlinico Universitario Agostino Gemelli—IRCCS, 00168 Roma, Italy; angela.rizzi@policlinicogemelli.it (A.R.); raffaella.chini01@gmail.com (R.C.); cristiano.caruso@policlinicogemelli.it (C.C.); 4 ENT Department, Rivoli Hospital, ASL TO3, 10098 Rivoli, Italy; rodolfomastrapasqua@gmail.com; 5 Unit of Chemistry, Biochemistry and Clinical Molecular Biology, Department of Laboratory and Hematological Sciences, Fondazione Policlinico Universitario Agostino Gemelli-IRCCS, Università Cattolica del Sacro Cuore, 00168 Roma, Italy; silvia.baroni@policlinicogemelli.it; 6 UOC Reumatologia, Università Cattolica del Sacro Cuore, Fondazione Policlinico Universitario Agostino Gemelli—IRCCS, 00168 Roma, Italy; augusta.ortolan@policlinicogemelli.it; 7 Department of Cardiovascular and Thoracic Sciences, Fondazione Policlinico Universitario Agostino Gemelli—IRCCS, 00168 Roma, Italy; matteo.bonini@policlinicogemelli.it (M.B.); francesca.cefaloni@unicatt.it (F.C.); cristina.boccabella@gmail.com (C.B.); francesco.lombardi@policlinicogemelli.it (F.L.); 8 Neuroradiology, Fondazione Policlinico Universitario Agostino Gemelli—IRCCS, 00168 Roma, Italy; marco.panfili@policlinicogemelli.it

**Keywords:** CRSwNP, biologics, functional endoscopic sinus surgery, systemic corticosteroids, intranasal corticosteroids, non-steroidal anti-inflammatory drugs, benralizumab, IL5

## Abstract

Background/Objectives: Benralizumab is a monoclonal antibody that targets the interleukin-5 receptor (IL-5Rα), leading to the rapid depletion of blood eosinophils. RCTs have demonstrated efficacy in patients with severe eosinophilic asthma (SEA). The aim of this study was to assess the efficacy of benralizumab on sinonasal outcomes in a real-life setting in patients with SEA and concomitant chronic rhinosinusitis with nasal polyps (CRSwNP). Methods: We included 25 patients (mean age: 57.47 years, range: 35–77, F/M = 12:13) who were prescribed 30 mg benralizumab every month for the first three administrations and then every 2 months. The primary endpoint was to evaluate changes in the SinoNasal Outcome Test-22 (SNOT-22) and nasal polyp score (NPS) over a 24-month treatment period. Secondary endpoints included measuring the effects on nasal obstruction and impaired sense of smell. Results: The mean NPS score decreased significantly from 5.11 ± 1.84 at baseline to 2.37 ± 1.96 at 24 months. The mean SNOT-22 decreased from 57 ± 15.30 at baseline to 26 ± 16.73 at 24 months. The SSIT-16 mean score improved with an increase in olfactory performance from 5.23 ± 2.58 at baseline to 7 ± 3.65 at 24 months. Moreover, 8/25 patients (32%) required rescue treatment with systemic steroids and 2 patients required endoscopic sinus surgery. Conclusions: While the improvement may not seem optimal at 12 months, a progressive enhancement was noted during the second year of treatment. Despite our data showing an improvement in quality of life and a reduction in the size of nasal polyps, no significant improvement in olfactory sensitivity was observed. In addition, in several patients, rescue treatments were required to maintain control of nasal and sinus symptoms. A careful risk–benefit assessment is therefore needed when deciding to continue treatment, weighing the potential for further improvement against the risks of complications. Such decisions should always be made in the context of a multidisciplinary team.

## 1. Introduction

Benralizumab is a humanized afucosylated monoclonal antibody produced in Chinese hamster ovary cells. It specifically targets the alpha subunit of the interleukin-5 receptor (IL-5Rα), which is predominantly found on human eosinophils and basophils. By binding to these IL-5Rα-expressing cells, benralizumab blocks IL-5 signaling and induces enhanced antibody-dependent cellular cytotoxicity, leading to the rapid and near-complete depletion of blood eosinophils and a reduction in basophil counts. Three Phase 3 randomized controlled trials—CALIMA [1], SIROCCO [2], and ZONDA [3]—have demonstrated benralizumab efficacy in severe eosinophilic asthma (SEA) patients. The recommended dosage of benralizumab is 30 mg, administered via subcutaneous injection every four weeks for the first three doses, followed by every eight weeks thereafter. The CALIMA [1] and SIROCCO [2] trials reported a significant reduction in asthma exacerbations, improved lung function, better asthma control, and enhanced quality of life. In the ZONDA trial [3], which focused on patients requiring maintenance oral corticosteroid (mOCS) therapy, the daily median OCS dose decreased by 50% compared to placebo, and the annual exacerbation rate (AER) decreased by 70%. 

Preliminary evidence suggested that benralizumab may be effective in sinonasal outcomes in patients with SEA and concomitant chronic rhinosinusitis with nasal polyps [4]. Canonica et al. [5] demonstrated the efficacy of benralizumab in improving SinoNasal Outcome Test 22 (SNOT-22) scores in patients with asthma and nasal polyps in a post hoc analysis of the ANDHI phase III-b trial, involving 153 patients with SEA and CRSwNP as a comorbidity. Real-world studies and case reports have consistently demonstrated the efficacy and safety of benralizumab in patients with severe eosinophilic asthma (SEA) and CRSwNP. Lombardo et al. [6] evaluated a cohort of 10 SEA patients with CRSwNP treated with benralizumab, reporting a marked improvement in endoscopic nasal polyp score (NPS), Lund–Mackay Score, and SNOT-22 after 24 weeks of treatment. Similarly, Bagnasco et al. [7] conducted a real-world analysis involving 34 patients with SEA and CRSwNP, confirming benralizumab’s effectiveness in reducing SNOT-22 scores, with 8 out of 26 patients (31%) regaining their sense of smell after six months of therapy.

In a small proof-of-concept study, benralizumab was shown to reduce baseline nasal polyp score (NPS) and improve symptoms, including the sense of smell, in severe CRSwNP patients [8]. This phase II randomized, double-blind, placebo-controlled trial, conducted over 20 weeks, demonstrated that benralizumab significantly improved endoscopic NPS, CT scores, SNOT-22, and UPSIT scores compared to baseline in patients with severe refractory CRSwNP, with at least one prior polypectomy.

The Phase 3 OSTRO study [9] evaluated the efficacy and safety of benralizumab in patients with CRSwNP who were symptomatic despite intranasal corticosteroid use and had a history of systemic corticosteroid use or nasal polyp surgery. In that trial, 413 patients with severe CRSwNP were enrolled and benralizumab significantly improved both NPS and nasal blockage scores compared to placebo (*p* ≤ 0.005). On the other hand, no significant differences were found in SNOT-22 scores, time to first nasal polyp surgery or systemic corticosteroid use, or time to first nasal polyp surgery between groups. There was nominal significance for improvement in sense of smell impairment (*p* = 0.003). The ongoing ORCHID trial [10] is currently evaluating the efficacy and safety of benralizumab in patients with severe CRSwNP, both with and without coexisting asthma.

Considering these premises, the aim of this study was to assess the efficacy of benralizumab on sinonasal outcomes in patients with severe eosinophilic asthma and concomitant CRSwNP in a real-life setting. The primary endpoint was to evaluate changes in the SNOT-22 scores and NPS over a 24-month treatment period with benralizumab. Secondary endpoints included measuring the effects of the drug on nasal obstruction, impaired sense of smell, and other primary symptoms of chronic rhinosinusitis.

## 2. Materials and Methods

### 2.1. Population and Study Design

This was a monocentric observational study in a real-life setting. We included 25 patients (mean age: 57.47 years; range: 35–77, F/M = 12:13) affected by severe eosinophilic asthma and CRSwNP who were prescribed 30 mg benralizumab every month for the first 3 administrations and then every 2 months by our pulmonologists and allergologists, and who were followed in a multidisciplinary setting for severe eosinophilic asthma and comorbid CRSwNP. As per current clinical practice, in CRSwNP patients, intranasal corticosteroids (INCS) were concomitantly prescribed. 

Sinonasal outcomes were monitored at the Rhinology Unit of the A. Gemelli Hospital Foundation IRCCS, UCSC in Rome, Italy, between February 2021 and February 2024. Benralizumab was prescribed by the pulmonologist and allergologist according to the Agenzia Italiana del Farmaco (AIFA) eligibility criteria [11,12,13], European Respiratory Society/American Thoracic Society guidelines and Global Initiative for Asthma (GINA) recommendations as an add-on treatment in patients with severe, uncontrolled eosinophilic asthma who had experienced at least two asthma exacerbations requiring systemic corticosteroids in the previous 12 months, despite optimized inhaled treatment. A blood eosinophil count of at least 300 cells/μL at the initiation of therapy was also required. Benralizumab was administered subcutaneously at a dose of 30 mg every 4 weeks for the first 3 doses, followed by every 8 weeks thereafter. 

The exclusion criteria for participation in this study included pregnancy, the presence of autoimmune diseases and/or concomitant immunosuppressive therapy including long term systemic steroids, non-compliance with continuous intranasal corticosteroid use, and history of radio-chemotherapy for cancer within the 12 months preceding treatment initiation. Informed consent regarding data privacy and the use of clinical information was obtained from all participants at the time of initial data collection. Clinical data were analyzed anonymously.

### 2.2. Methodology and Efficacy Outcomes

In routine clinical practice, following our institutional protocol, patients were assessed at multiple time points: at baseline (V0) prior to initiating biologic therapy, and during treatment at 6 months (V1), 12 months (V2), 18 months (V3), and 24 months (V4) following the first administration. At both baseline and follow-up visits, comprehensive evaluations were conducted, including endoscopic examination, quality of life assessments, nasal obstruction and olfaction measurements, and specific assessments of asthma symptom scores.

#### 2.2.1. Endoscopic Evaluation

The size of nasal polyps was assessed using the nasal polyp score (NPS), with each nasal cavity evaluated independently. The scores ranged from 0 to 4 per side, where 0 indicated the absence of polyps, 1 represented small polyps confined to the middle meatus without extending below the inferior border of the middle turbinate, 2 indicated polyps extending below the middle turbinate, 3 represented large polyps reaching the inferior turbinate or positioned medially to the middle turbinate, and 4 signified large polyps completely obstructing the nasal cavity. The total NPS was calculated by summing the scores from both nasal cavities [14].

#### 2.2.2. Quality of Life Assessment and VAS for Symptoms 

To assess quality of life, we employed the validated Italian version of the SNOT-22, which provides a total score ranging from 0 to 110. Scores below 20 indicated mild symptom severity. During the follow-up period, a change of 8.9 points in the SNOT-22 score was considered the minimal clinically important difference (MCID), based on prior research findings [15].

Symptom severity was evaluated using a 10 cm visual analog scale (VAS), where patients marked their symptom intensity on a horizontal line. The average score for each symptom was calculated by taking the mean of all individual scores for that symptom [16,17]. Specific VAS assessments were conducted for nasal obstruction, olfactory function, rhinorrhea, and facial pain.

#### 2.2.3. Olfactory Evaluation

The intensity of olfactory dysfunction (hyposmia) was assessed using a visual analog scale (VAS). Patients rated their olfactory ability on a 10 cm horizontal line, with the scale providing a continuum from no olfactory perception to normal olfactory function [17].

The Sniffin’ Sticks-16 Identification Test (SSIT-16) evaluates olfactory function by presenting 16 odors at a suprathreshold intensity. Patients are required to identify each odor from a set of four possible choices. The test yields a score ranging from 0 to 16, where 0 indicates no correct identifications and 16 denotes all odors identified accurately. Based on the scores, patients were categorized as anosmic (0–5), hyposmic (6–10), or normosmic (11–16) [17,18].

#### 2.2.4. Evaluation of Asthma Control 

The Asthma Control Test (ACT) is a Patient Reported Outcome (PRO) designed to evaluate various aspects of asthma management, including the frequency of shortness of breath and asthma symptoms, use of rescue medications, impact on daily activities, and overall self-assessment of asthma control. Scores on the ACT range from 5, indicating poor asthma control, to 25, representing complete control. Higher scores denote better asthma control, with a score above 20 suggesting well-controlled asthma. A difference of three points between individuals or over time is considered the minimal clinically important difference (MCID) [19,20,21,22].

### 2.3. Statistical Analysis

The data analysis was conducted using SPSS for Windows version 27 (IBM Corp, Chicago, IL, USA). The normality of continuous variables was assessed with the Shapiro–Wilk test, where *p*-values greater than 0.05 indicated normal distribution. For data with a normal distribution, paired t-tests were employed. For non-normally distributed data, the Mann–Whitney U-test was utilized. The results are presented as mean ± standard deviation (SD), with statistical significance determined at *p*-values less than 0.05. Comparisons were made between baseline data and measurements taken at various follow-up intervals (e.g., 6, 12, 18, and 24 months post-therapy). Outcomes were analyzed for all patients up to the 24-month mark. Multiple comparisons were adjusted using the Bonferroni correction (5x factor). Violin plots were generated using the R ggplot2 package via an online tool (https://www.statskingdom.com/violin-plot-maker.html, accessed on 13 July 2024).

## 3. Results

### 3.1. Baseline Characteristics of the Population

A total of 25 severe asthmatic patients (mean age: 57.47 ± 8.85 years; range: 35–77, F: 12, M: 13) with comorbid CRSwNP treated with benralizumab were included. All subjects underwent a 24-month observation period. Demographic and clinical data are reported in Table 1.

### 3.2. Efficacy of Benralizumab on Sino Nasal Outcomes 

**Figure 1:**
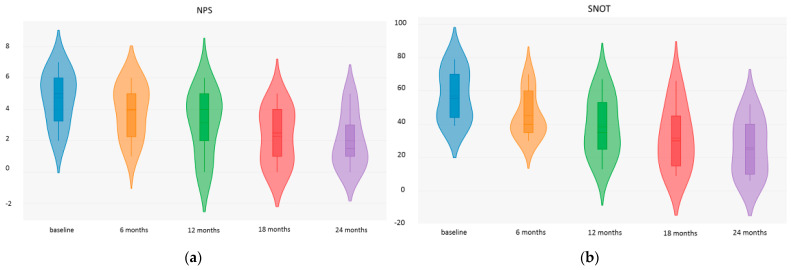
Nasal polyp endoscopic score (NPS) (**a**) and Sino-nasal Outcome Test−22 (SNOT−22) (**b**) fluctuation over time.

Benralizumab was shown to be effective in reducing NPS. The mean NPS decreased significantly from 5.11 ± 1.84 at baseline to 4.16 ± 1.65 at 6 months (*p* = 0.005), 3.41 ± 2.06 at 12 months (*p* = 0.005), 2.62 ± 1.92 at 18 months (*p* < 0.001), and 2.37 ± 1.96 at 24 months (*p* < 0.01) (Figure 1a).

In our series, we observed a slightly slow improvement in quality of life measured with several indicators. We observed an average reduction in SNOT-22 from 57 ± 15.30 at baseline to 45.28 ± 13.21 after 6 months (*p* = 0.015). The mean SNOT-22 further decreased to 38.5 ± 17.30 at 12 months (*p* < 0.001), 31.64 ± 18.89 at 18 months (*p* < 0.001), and 26 ± 16.73 at 24 months (*p* < 0.001).

We resumed the trend of all outcomes that we monitored in Table 2.

**Figure 2:**
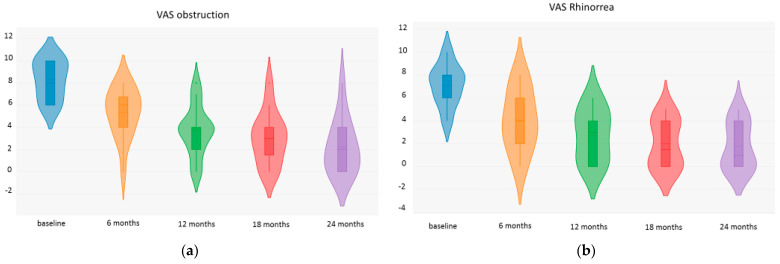

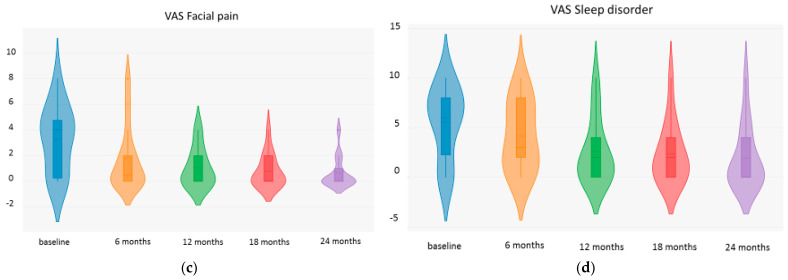
VAS nasal obstruction (**a**), rhinorrhea (**b**), facial pain (**c**), and sleep disturbance (**d**) fluctuations over time.

The VAS nasal obstruction was 8.28 ± 1.72 at baseline, 5.28 ± 2.30 at 6 months (*p* < 0.001), 3.71 ± 2.30 at 12 months (*p* < 0.001), 3 ± 2.38 at 18 months (*p* < 0.001), and 2.23 ± 2.45 at 24 months (*p* < 0.001). Figure 2 The VAS rhinorrhea was 7.13 + 1.81 at baseline, 4.07 + 2.64 at 6 months (*p* < 0.001), 2.71 + 2.27 at 12 months (*p* < 0.001), 2 + 2.04 at 18 months (*p* < 0.001), and 1.78 + 2.01 at 24 months (*p* < 0.001). We reported the fluctuation over time of the VAS for nasal obstruction, rhinorrhea, facial pain, and sleep disturbance in Figure 2.

Concerning the improvement in olfaction, we did not observe a significant improvement in the Sniffin’ Sticks−16 Identification test until 24 months of treatment. The SSIT-16 mean was 5.23 ± 2.58 at baseline, 5.46 ± 2.69 at 6 months of treatment (NS), 5.84 ± 2.70 at 12 months (NS), 6.30 ± 3.37 at 18 months (NS), and 7 ± 3.65 at 24 months (NS).

**Figure 3:**
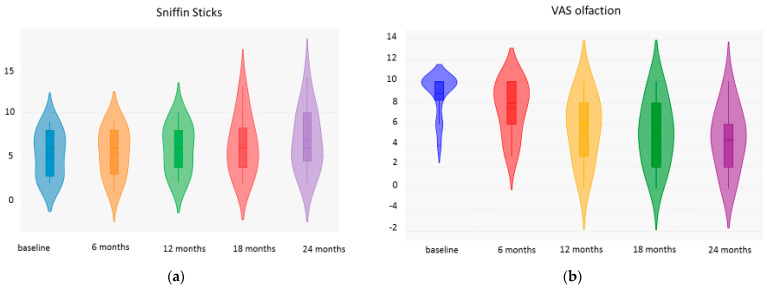
Sniffin Sticks (**a**) and VAS olfaction (**b**) fluctuations over time.

For what concerns the VAS olfaction we observed a slight improvement over 24 months. It was 8.86 + 1.96 at baseline, 7.50 + 2.50 at 6 months (NS), 5.64 + 3.08 at 12 months (*p* = 0.025), 5.21 + 3.09 at 18 months (*p* = 0.025), and 4.57 + 3.05 at 24 months (*p* = 0.015) (Figure 3).

### 3.3. Efficacy of Benralizumab on Disease Control in Terms of Need for OCSs and Surgery

The mean number of short courses of oral corticosteroids (OCSs) used in the year prior to baseline in our cohort was 2.11 ± 1.45. At 24 months, the mean number of systemic steroids required in the preceding year decreased to 0.9 ± 1.33 (*p* < 0.05). Nevertheless, over the entire 24−month treatment period, 8 out of 25 patients (32%) required rescue treatment with systemic steroids, mainly for improving sinonasal symptom control. 

Regarding surgical history before treatment, at baseline, 18 out of 25 patients (72%) had undergone at least one prior surgery for CRSwNP (Table 1). During the 24−month treatment period, two patients required endoscopic sinus surgery: one due to poor sinonasal disease control and the other due to a severe adverse event, specifically acute unilateral purulent ethmoid maxillary sinusitis with orbital abscess and vision loss in the right eye.

### 3.4. Efficacy of Benralizumab on Symptom Control of Asthma 

Regarding the impact of benralizumab on the symptom control of asthma, we observed a significant improvement in the Asthma Control Test (ACT) score throughout the treatment period. At baseline, the mean ACT score was 16 ± 5.41. This score showed a significant progressive increase at 6, 12, 18, and 24 months, with mean scores of 20.11 ± 5.20 (*p* = 0.045), 21.12 ± 3.79 (*p* < 0.001), 24 ± 1.52 (*p* < 0.001), and 24.14 ± 1.46 points (*p* < 0.001), respectively. Compared to baseline, the scores improved beyond the MCID and reached values greater than 19, consistently indicating well-controlled asthma after the first 6 months. 

### 3.5. Treatment Discontinuation and Safety of Benralizumab

Regarding adverse effects, benralizumab was well tolerated by 23 out of 25 patients in the study. In two cases, the treatment had to be discontinued due to severe adverse reactions. One patient experienced severe arthralgia, requiring rheumatologic evaluation and medical intervention. Another patient developed severe unilateral acute purulent ethmoid maxillary sinusitis with associated orbital cellulitis and abscess, resulting in vision loss in the right eye, and requiring urgent surgery. 

In two patients, benralizumab was discontinued due to the inadequate control of sinonasal symptoms and the patients were switched to alternative biologic treatment targeting both asthma and nasal polyps.

## 4. Discussion

Chronic rhinosinusitis with nasal polyps is a complex inflammatory disorder that significantly impacts patients’ quality of life and presents substantial challenges in therapeutic management [23]. The disease exhibits different phenotypes and endotypes, with eosinophilic inflammation being common in Western populations [24]. This condition is frequently associated with severe eosinophilic asthma (SEA), with more than 40% of severe asthmatics [25] and up to 60% of late-onset SEA patients suffering from CRSwNP [26]. The presence of eosinophilic inflammation in both conditions contributes to increased disease severity, treatment resistance, and recurrence, making CRSwNP a major asthma-treatable trait. In comorbid eosinophilic asthma and CRSwNP, eosinophils, under the action of type 2 cytokines including IL-5, significantly contribute to airway hyperresponsiveness, inflammation, epithelial cell damage, remodeling, and clinical exacerbations [27]. From a clinical point of view, tissue eosinophilia is frequently associated with extensive sinus disease, higher postoperative symptom scores, less improvement in both disease-specific and general QoL, and a higher polyp recurrence rate [28].

Traditional treatment options, including intranasal corticosteroids, saline irrigations, and systemic corticosteroids, are often supplemented by endoscopic sinus surgery in refractory cases. However, a subset of patients, identified as having “severe uncontrolled CRSwNP”, continue to experience persistent or recurrent symptoms despite medical and surgical interventions, underscoring the need for more effective therapeutic strategies. 

Given that the pathophysiology of CRSwNP is primarily driven by eosinophilic inflammation, involving T-helper cell 2 cytokines and IgE production [29], biological therapies with monoclonal antibodies—originally developed for conditions like asthma or atopic dermatitis, which share an underlying type 2 inflammatory pathway—can also be utilized for type 2 CRSwNP. These therapies target pivotal specific immunologic mediators of the inflammatory process, including anti-IL-4/IL-13 signaling (dupilumab), anti-IL-5 pathways (mepolizumab, benralizumab), and anti-IgE antibodies (omalizumab) [23].

Eosinophils play a crucial role in the development and persistence of nasal polyps, particularly through their involvement in inflammatory processes. The expression of chemoattractants like IL-5 and eotaxins promotes the migration of eosinophils to the upper airways, where they contribute to inflammation and tissue damage [30]. Eosinophils release cytotoxic granule proteins, such as eosinophil cationic protein (ECP) and eosinophil peroxidase (EPO), which can disrupt the extracellular matrix, induce oxidative stress, and cause cell cytotoxicity. These processes contribute to chronic tissue remodeling, a hallmark of nasal polyposis. Additionally, eosinophils are involved in the formation of extracellular traps (EETs), which have been detected in chronic rhinosinusitis with nasal polyps, further linking eosinophilic activity to the pathophysiology of this condition [31]. 

Benralizumab is an anti-eosinophilic antibody that has been shown to effectively reduce symptoms and exacerbations in patients with severe uncontrolled asthma characterized by eosinophilic inflammation. The SIROCCO [2] and CALIMA [1] Phase III studies involved patients aged 12 to 75 years with severe uncontrolled asthma, all of whom were receiving high-dose ICS and LABA therapy. Participants were randomized into three groups: (1) benralizumab 30 mg subcutaneously every 8 weeks, (2) benralizumab every 4 weeks, or (3) placebo. The results of these studies indicated that among various baseline factors (such as lung function assessed by FVC, use of oral corticosteroids, age at diagnosis, and frequency of exacerbations), the presence of nasal polyposis significantly influenced the clinical efficacy of benralizumab. Specifically, patients with nasal polyps experienced a greater reduction in exacerbation rates with benralizumab compared to placebo. Nasal polyps also had the most significant impact on improving FEV1 with benralizumab administered every 8 weeks versus placebo. Furthermore, the presence of nasal polyps was the most substantial baseline factor associated with a reduction in asthma symptom scores in the overall population. These findings led to the conclusion that the presence of nasal polyps was the most consistent predictor of clinical response to benralizumab, regardless of baseline blood eosinophil count. This is likely because nasal polyposis is associated with eosinophilic inflammation in the upper airways, which correlates with inflammation in the lower airways. Additionally, nasal polyposis is a predictor of asthma severity, particularly in patients with adult-onset asthma.

Growing evidence supports the benefits of long-term treatment with benralizumab on various nasal function outcomes in patients with asthma and chronic rhinosinusitis with nasal polyps (NPs). Recently, investigators of the RANS Study [32] conducted a retrospective, multi-country observational study involving 233 patients to explore NP and asthma outcomes. During the follow-up, 67.6% (71/105) of patients showed a significant improvement in the SinoNasal Outcome Test-22 (SNOT-22) total score, with a reduction of ≥8.9 points. Additionally, 49.1% (28/57) of patients achieved a clinically meaningful reduction in the total NP score (NPS) (reduction of ≥1 point).

An Italian retrospective, multicenter, and observational study further evaluated the therapeutic effects of benralizumab on the upper and lower airways in patients with severe eosinophilic asthma (SEA) over two years, focusing on the induction of sustained clinical remission. The study enrolled 164 patients with SEA between September 2019 and March 2023 and established the following a priori criteria for response to benralizumab: SNOT-22 < 30 and NP relapses = 0 after two years of biological therapy. Among the 82 patients with SEA and concomitant NPs, 33 (40.2%) demonstrated a persistent improvement in nasal function scores (SNOT-22 < 30, NP recurrence = 0) after 24 months of treatment with benralizumab [33].

In this study, we reported our experience in real life on 25 patients with severe asthma and comorbid chronic rhinosinusitis with nasal polyps (CRSwNP) treated with benralizumab and followed at our institution for at least 24 months. In our experience, benralizumab has proven effective in improving asthma symptoms, as confirmed by the significant improvement in the ACT score from 16 to 24.14 over 24 months, indicating substantial progress in managing asthma symptoms. Furthermore, our data demonstrate that benralizumab gradually and slowly improves sinonasal outcomes in treated patients. Concerning the sinonasal outcomes, the most significant improvements were observed in the nasal polyp score (NPS) and visual analog scale (VAS) for nasal obstruction. Indeed, benralizumab substantially reduced the NPS from 5.11 at baseline to 2.37 after 24 months, while the VAS for nasal obstruction significantly decreased from 8.28 at baseline to 2.23 at 24 months. It should be underlined that 2 patients out of 25 underwent surgery, contributing to the significant reduction in the scores.

A moderate improvement over time was also observed in the SinoNasal Outcome Test-22 (SNOT-22) scores. The SNOT-22 score, reflecting quality of life, improved from a baseline of 57 to 26 after 24 months. 

However, the drug proved to be ineffective in improving olfactory function, both in semi-objective tests and based on symptom scores. Specifically, the mean SSIT-16 score improved from 5.23 ± 2.58 at baseline to 5.84 ± 2.70 at 12 months and to 7 ± 3.65 at 24 months. This improvement is considered clinically insignificant, as the mean score moved from a state of anosmia to one of severe hyposmia. Similarly, the olfactory VAS decreased from 8.86 ± 1.96 at baseline to 5.64 ± 3.08 at 12 months and to 4.57 ± 3.05 at 24 months, remaining greater than 3 over the entire time of observation. 

Regarding the need for rescue treatments, it is important to note that 40% of the patients required rescue treatment due to the inadequate control of sinonasal symptoms. In detail, 10 out of 25 patients required such treatments, with 8 requiring oral corticosteroids and 2 requiring surgery. Our data also highlight the need for close clinical monitoring of patients treated with benralizumab for asthma by ENT specialists to assess nasal symptom control and prevent the potential risk of dangerous exacerbations.

The strength of our study lies in its real-life context, extended observation period, multidisciplinary management, and rigorous follow-up schedule. Therapeutic outcomes were monitored throughout the 2 years of treatment, allowing us to observe the gradual benefits of benralizumab on sinonasal outcomes in a real-world setting. In line with previous studies [34], we observed a significant improvement in both SNOT-22 and NPS scores. However, unlike these studies, our study did not show a significant improvement in olfactory function, as documented by the Sniffin’s Sticks test scores.

However, there are some limitations to consider. This study was conducted at a tertiary referral center, reporting on our initial cohort of patients, which may include those with the most severe and challenging cases of CRSwNP. Furthermore, the rescue treatments used in real life may have influenced the results observed at 24 months. Future studies involving non-academic patient populations will help determine whether different outcomes might emerge on a larger national scale. Additionally, since benralizumab was primarily prescribed for asthma rather than for nasal polyps, and given the relatively small number of patients recruited, these factors may limit the generalizability of our findings.

## 5. Conclusions

In conclusion, we observed an improvement in sinonasal outcomes in patients with severe eosinophilic asthma treated with benralizumab over a 24-month period. While the improvement may not seem optimal at 12 months, a slow and progressive enhancement in outcomes was noted during the second year of treatment. The authors of this article believe that the decision to continue treatment in cases of poor nasal symptom control should be made based on a careful risk–benefit assessment. On the one hand, there are the benefits achieved in asthma control and the potential for further improvement in the second year of treatment. On the other hand, these benefits must be weighed against the risks of complications associated with suboptimal nasal–sinus control, systemic steroid use, and the impact of symptoms on the patient’s quality of life. Therefore, such decisions should always be made in the context of a multidisciplinary team and taking into account the preferences of the patient. 

## Figures and Tables

**Table 1:** Demographic and clinical data of the cohort (*n* = 25 patients).

	Number or Mean Score ± Standard Deviation	(%)
Number of patients	25	
Age in years (mean age)	57.47 ± 8.85	
Male	13/25	52%
Female	12/25	48%
BMI (kg/m^2^)	25.03 ± 3.17	
Evidence of type 2 inflammation		
SEA	25/25	100%
NSAIDs intolerance	8/25	32%
Peripheral blood eosinophils (>300 cell/μL)	25/25	100%
IgE (>100 IU/mL)	17/25	68%
Mean CT Lund Mackay score	18.3 ± 4.1	
Mean SNOT-22	57 ± 15.30	
Mean NPS	5.11 ± 1.84	
Mean Sniffin’ Sticks Identification test score	5.23 ± 2.58	
ACT	16 ± 5.41	
Mean number of OCS bursts in the previous year	2.11 ± 1.45	
Previous ESS surgery for CRSwNP		
ESS = 0	7/25 (28%)	
ESS = 1	8/25 (32%)	
ESS > 1	10/25 (40%)	

Abbreviations. NSAIDs: non-steroidal anti-inflammatory drugs; IgE: Immunoglobulin-E; CT: computerized tomography; SNOT-22: sinonasal outcome test-22; NPS: nasal polyp score; OCS: oral corticosteroids; ESS: endoscopic sinus surgery.

**Table 2:** Patients’ clinical outcomes during treatment period.

	Baseline	6 Months	12 Months	18 Months	24 Months
Mean SNOT-22	57 ± 15.30	45.28 ± 13.21	38.5 ± 17.30	31.64 ± 18.89	26 ± 16.73
Mean NPS	5.11 ± 1.84	4.16 ± 1.65	3.41 ± 2.06	2.62 ± 1.92	2.37 ± 1.96
Mean Sniffin’Sticks-16 IT	5.23 ± 2.58	5.46 ± 2.69	5.84 ± 2.70	6.30 ± 3.37	7 ± 3.65
Mean eosinophilic blood count	650.22 ± 222.51	0	0	0	0
VAS olfaction	8.86 ± 1.96	7.50 ± 2.50	5.64 ± 3.08	5.21 ± 3.09	4.57 ± 3.05
VAS obstruction	8.28 ± 1.72	5.28 ± 2.30	3.71 ± 2.30	3 ± 2.38	2.23 ± 2.45
VAS rhinorrhea	7.13 ± 1.81	4.07 ± 2.64	2.71 ± 2.27	2 ± 2.04	1.78 ± 2.01
ACT	16 ± 5.41	20.11 ± 5.20	21.12 ± 3.79	24 ± 1.52	24.14 ± 1.46

Abbreviations: SNOT-22: sinonasal outcome test-22; NPS: nasal polyp score; VAS: visual analog scale.

## Data Availability

The data presented in this study are available on reasonable request from the corresponding author.
